# A Leucyl-tRNA Synthetase Inhibitor with Broad-Spectrum Antimycobacterial Activity

**DOI:** 10.1128/AAC.02420-20

**Published:** 2021-04-19

**Authors:** Uday S. Ganapathy, Rubén González del Rio, Mónica Cacho-Izquierdo, Fátima Ortega, Joël Lelièvre, David Barros-Aguirre, Marissa Lindman, Véronique Dartois, Martin Gengenbacher, Thomas Dick

**Affiliations:** a Center for Discovery and Innovation, Hackensack Meridian Health, Nutley, New Jersey, USA; b Global Health R&D, GlaxoSmithKline, Tres Cantos, Spain; c Department of Medical Sciences, Hackensack Meridian School of Medicine, Nutley, New Jersey, USA; d Department of Microbiology and Immunology, Georgetown University, Washington, DC, USA

**Keywords:** benzoxaborole, EC/11770, *Mycobacterium abscessus*, NTM, nontuberculous mycobacteria, leucyl-tRNA synthetase

## Abstract

Global infections by nontuberculous mycobacteria (NTM) are steadily rising. New drugs are needed to treat NTM infections, but the NTM drug pipeline remains poorly populated and focused on repurposing or reformulating approved antibiotics. We sought to accelerate *de novo* NTM drug discovery by testing advanced compounds with established activity against Mycobacterium tuberculosis. 3-Aminomethyl 4-halogen benzoxaboroles, a novel class of leucyl-tRNA synthetase inhibitors, were recently discovered as active against M. tuberculosis. Here, we report that the benzoxaborole EC/11770 is not only a potent antitubercular agent but is active against the M. abscessus and M. avium complexes. Focusing on M. abscessus, which causes the most-difficult-to-cure NTM disease, we show that EC/11770 retained potency against drug-tolerant biofilms *in vitro* and was effective in a mouse lung infection model. Resistant mutant selection experiments showed a low frequency of resistance and confirmed leucyl-tRNA synthetase as the target. This work establishes the benzoxaborole EC/11770 as a novel preclinical candidate for the treatment of NTM lung disease and tuberculosis and validates leucyl-tRNA synthetase as an attractive target for the development of broad-spectrum antimycobacterials.

## Abstract (precis)

Global infections by nontuberculous mycobacteria (NTM) are steadily rising. New drugs are needed to treat NTM infections, but the NTM drug pipeline remains poorly populated and focused on repurposing or reformulating approved antibiotics.

## INTRODUCTION

Nontuberculous mycobacteria (NTM) are a group of opportunistic pathogens belonging to the same genus as Mycobacterium tuberculosis, the causative agent of tuberculosis (TB) (1). Like M. tuberculosis, NTM pathogenesis often manifests as lung disease, requiring lengthy treatment with multidrug regimens. But while global TB cases have decreased in recent years, global NTM cases have steadily risen (2), highlighting the unique treatment challenges that these infections pose.

NTM lung infections are primarily caused by members of the M. abscessus and M. avium complexes. Despite similarities to M. tuberculosis, these NTM species display notable differences in their pathogenesis due to their expression of unique surface lipids, adaptation to both host and environmental niches, and acquisition of novel virulence strategies (3). While pulmonary infections are most common, M. abscessus and M. avium pathogenesis can also manifest as severe disseminated disease in immunocompromised individuals (4). M. abscessus and M. avium also exhibit intrinsic drug resistance to many classes of antibiotics typically administered to treat TB (5–7). The situation is most severe for M. abscessus infections, which are resistant to all first-line TB drugs and for which no curative treatment is available. As a result, the drug regimens for NTM lung disease are markedly different from the standard four-drug TB regimen and vary by species (8–10). For infections caused by M. avium complex, combination therapy with a macrolide (clarithromycin or azithromycin) and two TB drugs (ethambutol and rifampin) is recommended (10). Treatment of M. abscessus lung disease, however, involves a combination of a macrolide with parenterally administered antibiotics, typically an aminoglycoside and either imipenem, cefoxitin, or tigecycline as a third drug. While the potency of macrolides against M. abscessus can be limited by *erm41*-mediated inducible drug resistance (11), these compounds can still provide beneficial immunomodulatory effects such as reducing airway secretion to promote mucociliary clearance (12, 13). The fact that M. abscessus chemotherapy requires intravenous drug administration is another complicating factor not encountered in M. avium treatment, in which all drugs can be administered orally. In both cases, treatment typically lasts 18 to 24 months, produces severe drug side effects, and drives acquired drug resistance. Worst of all, treatment outcomes for NTM lung disease remain poor with cure rates averaging 60% for M. avium infections and 50% for M. abscessus infections (14, 15).

Given the poor performance of current NTM treatment regimens, new drugs are urgently needed to combat the rise in NTM infections (16). Ideally, new NTM drugs would have novel targets and mechanisms of action that can overcome both intrinsic and acquired drug resistance (17). The introduction of new drugs to NTM drug regimens could shorten treatment times, reducing adverse side effects and opportunity for acquired drug resistance. Unfortunately, the current NTM drug pipeline is sparsely populated and mostly focused on repurposing or reformulating approved antibiotics (https://clinicaltrials.gov and reference 17). *De novo* drug discovery campaigns remain largely absent due to their higher attrition rates compared to those of repurposing strategies, making them slower and costlier endeavors.

We have recently devised a two-part strategy to accelerate *de novo* NTM drug discovery. First, we selectively screen TB actives for anti-NTM activity. Since M. avium and M. abscessus are genetically related to M. tuberculosis, the likelihood that TB actives have homologous targets in these species will be greater. Indeed, screening TB actives yields a higher hit rate than screening a random compound collection (18). Second, we prioritize the screening of advanced compounds. With established pharmacokinetics (PK) and tolerability, advanced compounds circumvent several critical hurdles in *de novo* drug discovery, allowing them to move rapidly from *in vitro* NTM actives to lead compounds with demonstrated *in vivo* efficacy (19). With this strategy in mind, we sought to identify drug classes with novel bacterial targets and demonstrated activity against M. tuberculosis. The screening of advanced compounds from these drug classes would offer a shorter path to the discovery of new NTM drugs.

Aminoacyl-tRNA synthetases (aaRSs) are enzymes that correctly attach each amino acid to its cognate tRNA molecule, enabling proper translation of the genetic code (20). Given this critical role in protein biosynthesis, aaRSs represent potential antibiotic targets. While the organization of aaRS catalytic domains into two structurally distinct classes is conserved across all domains of life, there remain substantial structural differences between the aaRS catalytic domains of prokaryotes and those of eukaryotes (21). Therefore, a pathogen-specific aaRS inhibitor could be developed that does not target the human host enzyme. Mupirocin, the first clinically approved aaRS inhibitor, inhibits the aminoacylation activity of isoleucyl-tRNA synthetase (IleRS) by competing with isoleucine for binding to the enzyme’s catalytic domain (22, 23). While mupirocin is most active against Gram-positive bacteria, it is inactive against mycobacteria (24). Furthermore, mupirocin has poor bioavailability, restricting its use to topical treatment of skin infections and rendering this class of aaRS inhibitor unsuitable for the treatment of mycobacterial lung infections.

Benzoxaboroles are a class of boron-heterocyclic compounds that were first described as having potent antifungal activity (25). Saccharomyces cerevisiae mutants resistant to benzoxaborole AN2690 had mutations in the gene encoding leucyl-tRNA synthetase (LeuRS), suggesting that benzoxaboroles are aaRS inhibitors (26). In addition to a catalytic domain, several aaRSs contain an editing domain that can hydrolyze incorrectly charged tRNAs, providing a critical proofreading function that ensures fidelity of the genetic code (27). Intriguingly, all the LeuRS mutations identified in the S. cerevisiae AN2690-resistant mutants mapped to the editing domain, indicating that benzoxaboroles and mupirocin have different mechanisms of action (26). Crystallographic studies revealed that the oxaborole ring of AN2690 enables the formation of an AN2690-tRNA^Leu^ adduct that binds to the LeuRS editing domain. This oxaborole tRNA trapping (OBORT) mechanism efficiently inhibits protein synthesis by blocking leucyl-tRNA^Leu^ synthesis (26).

**FIG 1:**
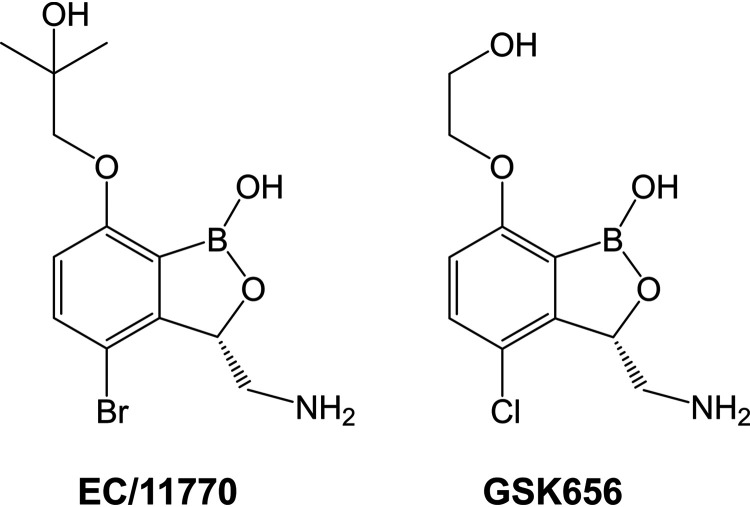
Structures of EC/11770 and GSK656.

Following a screening campaign by GlaxoSmithKline, 3-aminomethyl 4-halogen benzoxaboroles were identified as active against M. tuberculosis (28). Benzoxaborole-resistant mutants also carried mutations in the LeuRS editing domain, validating LeuRS as a TB drug target (28). Additional structure-activity relationship studies around the benzoxaborole scaffold led to the discovery of GSK3036656 (GSK656, Fig. 1), which had attractive *in vivo* PK and demonstrated efficacy in a mouse model of TB infection (29). In 2019, a first-time-in-human study demonstrated the safety and tolerability of GSK656 (30), furthering the development of this benzoxaborole as a promising new TB drug. Given the antitubercular activity of GSK656, we asked whether this compound and its close analog EC/11770 (Fig. 1) are active against NTM. Surprisingly, we found that GSK656 showed a restricted NTM spectrum, whereas EC/11770 displayed broad anti-NTM activity. Through detailed *in vitro* and *in vivo* profiling, we have established EC/11770 as a novel preclinical candidate for the treatment of NTM lung disease.

## RESULTS

### EC/11770 is active against M. abscessus and M. avium *in vitro*.

GSK656 and its close analog EC/11770 (Fig. 1) have potent activity against M. tuberculosis (MIC values of 0.08 μM and 0.56 μM, respectively; Table 1) (29). Therefore, we asked whether these compounds have activity against M. abscessus and M. avium in Middlebrook 7H9 medium (7H9). For these experiments, we used two recent clinical isolates of these NTM species: M. abscessus Bamboo (subsp. *abscessus*) (31) and M. avium 11 (subsp. *hominissuis*) (32). While GSK656 was active against M. abscessus Bamboo, this compound had no activity against M. avium 11 (Table 1). Surprisingly, EC/11770 was active against both M. abscessus Bamboo and M. avium 11 (Table 1). Since composition of the medium and the presence of detergents can affect potency (33, 34), we measured the MIC of EC/11770 in cation-adjusted Mueller-Hinton (CAMH) broth, which is the clinical standard for antibiotic susceptibility testing, has a different carbon source composition from 7H9, and lacks detergent (35). EC/11770 retained its potency against M. abscessus Bamboo in CAMH (Table 1). Thus, the benzoxaborole EC/11770 demonstrated dual activity against M. abscessus and M. avium screening strains, and this activity was culture medium independent.

**TABLE 1:** Activities of EC/11770 and GSK656 against M. tuberculosis, M. abscessus, and M. avium^*a*^

Strain	Medium	MIC (μM)^*b*^		
		CLR	EC/11770	GSK656
M. tuberculosis H37Rv ATCC 27294	7H9	ND^*c*^	0.56	0.08^*d*^
M. abscessus Bamboo	7H9	0.23	1.2	0.27
M. abscessus Bamboo	CAMH	0.07	0.7	ND
M. avium 11	7H9	0.30	4.0	>50

a MIC values are the mean of two independent experiments.

b CLR, clarithromycin; ND, not determined.

c MIC of rifampin against M. tuberculosis H37Rv was 0.66 μM.

d MIC value of GSK656 against M. tuberculosis H37Rv is from published literature (29).

### EC/11770 has broad-spectrum anti-NTM activity.

As EC/11770 was active against M. abscessus Bamboo (Table 1), we asked whether EC/11770 is active against all three subspecies of the M. abscessus complex (subsp. *abscessus*, subsp. *massiliense*, and subsp. *bolletii*), which are known to exhibit differences in antibiotic susceptibility (8, 36). EC/11770 showed comparable potency against culture collection reference strains for all three M. abscessus subspecies (Table 2). Furthermore, EC/11770 retained its activity against a panel of clinical isolates of M. abscessus that cover the M. abscessus complex (Table 2) (37). EC/11770 was also active against M. abscessus subsp. *abscessus* K21, a clinical isolate used in our M. abscessus mouse infection model (Table 2) (38). The M. avium complex consists of 12 distinct species, of which three (M. avium, M. intracellulare, and M. chimaera) are the most common causative agents of NTM lung disease (10, 39). Given EC/11770’s activity against M. avium 11 (Table 1), we asked whether this compound has activity against other members of the M. avium complex. Indeed, EC/11770 inhibited the growth of culture collection reference strains of M. intracellulare and *M. chimaera* (Table 2). Thus, EC/11770 displayed broad-spectrum antimycobacterial activity that targets M. tuberculosis and the M. abscessus and M. avium complexes. As M. abscessus NTM disease is the most difficult to cure, we focused subsequent analyses of EC/11770 on this mycobacterial species.

**TABLE 2:** Broad-spectrum antimycobacterial profiling of EC/11770^*a*^

		MIC (μM)^*b*^	
Strain	Strain type	CLR	EC/11770
M. abscessus Bamboo	Clinical isolate, screening strain	0.23	1.2
M. abscessus subsp. *abscessus* ATCC 19977	Culture collection reference strain	0.90	0.70
M. abscessus subsp. *massiliense* CCUG 48898T	Culture collection reference strain	0.19	0.71
M. abscessus subsp. *bolletii* CCUG 50184T	Culture collection reference strain	2.5	1.3
M. abscessus subsp. *abscessus* M9	Clinical isolate	0.73	0.49
M. abscessus subsp. *abscessus* M199	Clinical isolate	2.7	0.93
M. abscessus subsp. *abscessus* M337	Clinical isolate	0.90	0.50
M. abscessus subsp. *abscessus* M404	Clinical isolate	0.20	0.52
M. abscessus subsp. *abscessus* M422	Clinical isolate	0.65	0.33
M. abscessus subsp. *bolletii* M232	Clinical isolate	0.95	0.67
M. abscessus subsp. *bolletii* M506	Clinical isolate	0.28	0.48
M. abscessus subsp. *massiliense* M111	Clinical isolate	0.24	0.95
M. abscessus subsp. abscessus K21	Clinical isolate, infection model	0.40	0.60
M. avium 11	Clinical isolate, screening strain	0.30	4.0
M. intracellulare ATCC 13950	Culture collection reference strain	0.15	0.37
M. chimaera CCUG 50989T	Culture collection reference strain	0.19	1.7

a MIC values are the mean of two independent experiments.

b CLR, clarithromycin.

### EC/11770 is bacteriostatic against M. abscessus *in vitro*.

Benzoxaboroles lack bactericidal activity against M. tuberculosis (28), making them bacteriostatic compounds. We asked whether EC/11770 is also bacteriostatic against M. abscessus by determining the MIC and minimal bactericidal concentration (MBC) of this compound against planktonic bacteria growing in culture tubes (as opposed to the wells of 96-well plates) (40). Under these conditions, clarithromycin inhibited the growth of M. abscessus Bamboo (MIC = 0.28 μM) but had no bactericidal activity (MIC > 100 μM) (Table 3), consistent with the bacteriostatic profile of macrolides against this bacterium (40). Similarly, EC/11770 had growth inhibitory activity against M. abscessus Bamboo (MIC = 3 μM) but was not bactericidal (MBC > 100 μM) (Table 3). Consistent with M. tuberculosis potency data (28), these results demonstrated that benzoxaboroles are also bacteriostatic against NTM like M. abscessus.

**TABLE 3:** Growth inhibitory and bactericidal activity of EC/11770 against planktonic and biofilm M. abscessus^*a*^

	MIC (μM)^*b*^		MBC (μM)	
	CLR	EC/11770	CLR	EC/11770
Planktonic	0.28	3.0	>100	>100
Biofilm	1.6	3.1	>100	50

a MIC and MBC values are the mean of two independent experiments.

b CLR, clarithromycin.

### EC/11770 inhibits the growth of M. abscessus biofilms.

M. abscessus forms biofilms that are tolerant to several classes of antibiotics active against planktonic cultures of the pathogen (40–42). We therefore assessed the potency of EC/11770 in an *in vitro* M. abscessus biofilm growth assay (40). Similar to clarithromycin, EC/11770 inhibited M. abscessus biofilm growth but did not have bactericidal activity against M. abscessus in established biofilms (Table 3). These results are consistent with the bacteriostatic activity of both compounds against M. abscessus in culture tubes (Table 3). However, while the MIC of clarithromycin increased 5- to 6-fold against M. abscessus biofilms, EC/11770 was as active against the biofilm and planktonic forms of the bacterium (Table 3). Thus, EC/11770 retained the potent growth inhibitory activity it displays in broth culture against M. abscessus biofilms.

### EC/11770 targets M. abscessus leucyl-tRNA synthetase LeuRS.

Benzoxaboroles were first discovered as inhibitors of LeuRS in fungi (26). In both M. tuberculosis and M. smegmatis, benzoxaborole-resistant mutants harbored point mutations in the gene encoding LeuRS (28), which has a homolog in M. abscessus (*leuS*, *MAB_4923c*). To determine whether LeuRS is the target of EC/11770 in M. abscessus, we selected for EC/11770-resistant mutants of M. abscessus Bamboo (Table 4). Based on two independent selections, we calculated a frequency of resistance to EC/11770 of 3.9 × 10^−8^/CFU, which was significantly lower than that reported for benzoxaboroles in M. tuberculosis (3.9 × 10^−6^ to 4.6 × 10^−6^/CFU) (28). MIC profiling of five EC/11770-resistant mutants (RM1-5) showed high level resistance to EC/11770 but no change in susceptibility to clarithromycin (Table 4). The EC/11770 MIC for two of the mutants (RM3 and RM5) was between 60 and 75 μM, while the remaining mutants’ EC/11770 MIC was greater than 100 μM. Sequencing of *leuS* revealed that all five resistant strains carried a single missense mutation in the LeuRS editing domain (residues V292 to K502), consistent with the OBORT mechanism of benzoxaboroles (Table 4 and Fig. S1) (26). RM1 carried an A428P substitution, a residue that was mutated in previously reported M. smegmatis benzoxaborole-resistant mutants (A to T) (28). RM4 had a T327R mutation, which maps to a threonine residue that was mutated in an S. cerevisiae benzoxaborole-resistant mutant (T319I) (26). The two other LeuRS missense mutations in the editing domain (K502E and V417M) were novel (26, 28). One of these novel missense mutations, V417M, was isolated twice from independent selection experiments (RM3 and RM5, Table 4). We conclude that EC/11770’s anti-NTM activity is mediated by targeting the editing domain of LeuRS as previously described for other benzoxaboroles (26, 28).

**TABLE 4:** Characterization of M. abscessus EC/11770-resistant mutants^*a*^

Strain	Batch	MIC (μM)^*b*^		
		CLR	EC/11770	LeuS mutations
M. abscessus Bamboo	0.31	0.9	None
RM1	1	0.36	>100	LeuS A428P
RM2	1	0.38	>100	LeuS K502E
RM3	1	0.43	62	LeuS V417M
RM4	2	0.42	>100	LeuS T327R
RM5	2	0.46	75	LeuS V417M

a MIC values are the mean of two independent experiments.

b CLR, clarithromycin.

### Pharmacokinetic properties of EC/11770.

**FIG 2:**
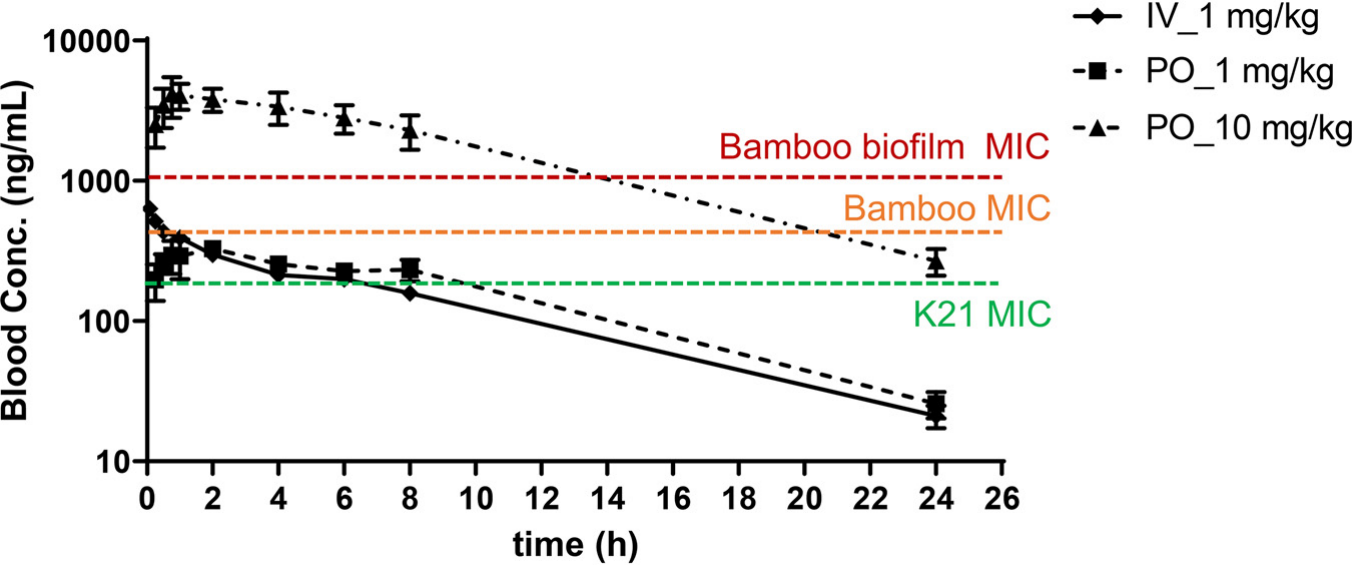
Blood concentration versus time profiles for EC/11770. Whole blood concentrations versus time profiles of EC/11770 after intravenous (IV) administration of 1 mg/kg and oral (PO) administration of 1 and 10 mg/kg. MIC values of M. abscessus Bamboo and K21 planktonic cultures (Table 2) and M. abscessus Bamboo biofilms (Table 3) are plotted.

EC/11770 exhibited attractive physicochemical properties leading to high solubility and permeability (Table 5). The low intrinsic clearance observed in mouse and human microsomes (<0.5 ml/min/g tissue) predicted the very low *in vivo* clearance obtained in mice: intravenous administration of 1 mg/kg saline solution resulted in an *in vivo* clearance rate of 5 ml/min/kg, corresponding to approximately 5% of the liver blood flow in mice (90 to 126 ml/min/kg) (Table 5). This low *in vivo* clearance, combined with a moderate volume of distribution, translated into a high average half-life in mice of 5.3 h (Fig. 2 and Table 5). After oral administration of EC/11770 at 1 mg/kg or 10 mg/kg, a high oral bioavailability was obtained for both doses (100%) (Fig. 2 and Table 5). These results are consistent with EC/11770’s good solubility, permeability, and low *in vivo* clearance (Table 5). EC/11770’s low *in vivo* clearance also minimizes the oral first-pass effect (Table 5). Additionally, the compound presented a reasonably linear pharmacokinetic behavior between the two oral doses: drug exposure increased proportionally with the increase in dosage, as reflected in the similar dose-normalized exposures obtained for both dosages (Table 5, dose-normalized area under the concentration-time curve, or DNAUC). Using these PK results and the potency data, we determined that 10 mg/kg would achieve 100% time above MIC in M. abscessus planktonic cultures and approximately 60% time above M. abscessus biofilm MIC (Fig. 2) (EC/11770 is known to be tolerated at doses up to 100 mg/kg in TB efficacy studies).

**TABLE 5:** Physicochemical and pharmacokinetic properties of EC/11770^*a*^

Parameter	Value
CLND solubility (μM)	349
ChromLogD pH 7.4	0.97
AMP pH 7.4 (nm/sec)	275
Mouse and human hepatic microsomes stability (*in vitro*)	
Mouse *in vitro* CL_int_ (ml/min/g tissue) Human *in vitro* CL_int_ (ml/min/g tissue)	<0.5 <0.5
Mouse pharmacokinetic parameters (*in vivo*)^*b*^	
Intravenous administration, 1 mg/kg	
*In vivo* CL (ml/min/kg) V_ss_ (liter/kg) *t*_1/2_ (h) AUC_inf_ (ng h/ml)	5.0 (0.1) 2.4 (0.1) 5.3 (0.3) 3,338 (79)
Oral administration, 1 mg/kg & 10 mg/kg	
*C*_max__1 mg/kg (ng/ml) *C*_max__10 mg/kg (ng/ml) *T*_max__1 mg/kg (h) *T*_max__10 mg/kg (h) AUC_0–24__1 mg/kg (ng h/ml) AUC_0–24__10 mg/kg (ng h/ml) DNAUC_0–24__1 mg/kg (ng h/ml per mg/kg) DNAUC_0–24__10 mg/kg (ng h/ml per mg/kg) %F_1 mg/kg %F_10 mg/kg	341 (51) 4,393 (1,260) 1.0−2.0 0.75−2.0 3,556 (179) 40,532 (9,083) 3,556 (179) 4,053 (908) ∼100 ∼100

a CLND solubility, aqueous solubility via chemiluminescent nitrogen detection; AMP, artificial membrane permeability; CL_int_, intrinsic clearance; *in vivo* CL, *in vivo* clearance; V_ss_, volume of distribution at steady state; *t*_1/2_, half-life; AUC_inf_, area under the concentration-time curve extrapolated to infinite; *C*_max_, highest concentration of drug in the blood; *T*_max_, time taken to reach *C*_max_; AUC_0–24_, area under the concentration-time curve from time 0 to 24 h; DNAUC, dose-normalized area under the concentration-time curve; %F, bioavailability.

b Average (SD). *T*_max_ is expressed as a range of values. %F is expressed as a percentage.

### EC/11770 is active against M. abscessus *in vivo*.

**FIG 3:**
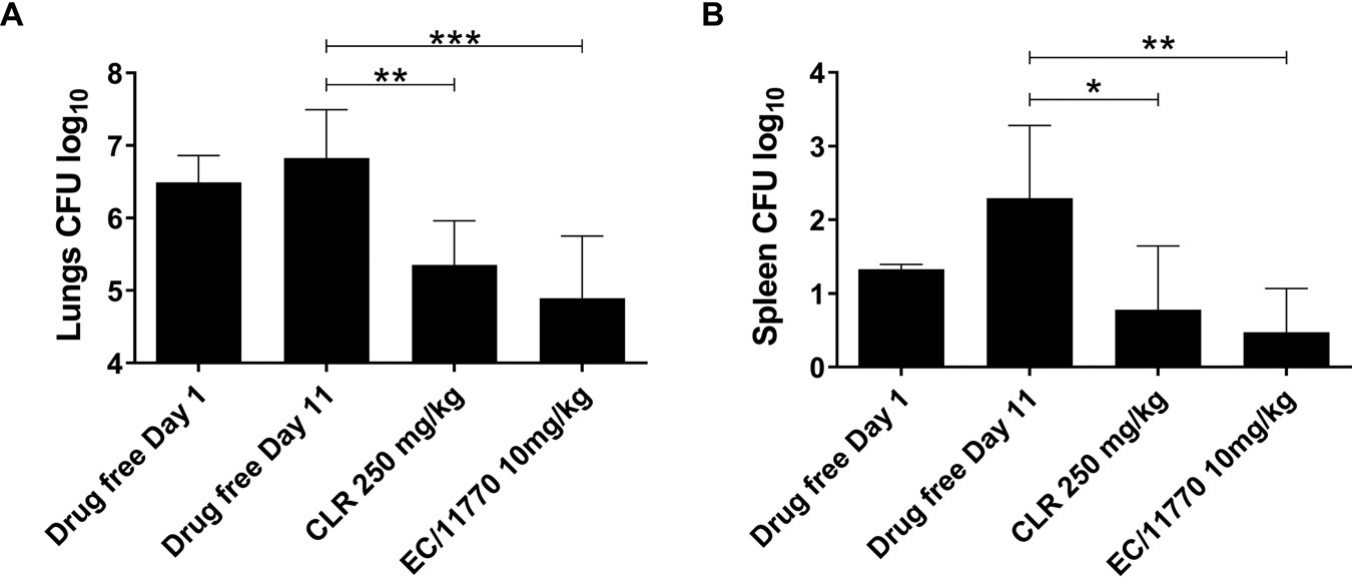
EC/11770 is active against M. abscessus *in vivo*. Lung CFU (A) and spleen CFU (B) from NOD SCID mice 1 day after intranasal infection with *Mab* (drug-free day 1) and following daily oral administration of drug-free vehicle, clarithromycin (CLR), or EC/11770 for 10 days (day 11). Data represent the mean plus standard deviation of six mice per treatment group. Statistical significance of the results was analyzed by one-way analysis of variance (ANOVA) multicomparison and Tukey’s posttest (*, *P* < 0.05; **, *P* < 0.01; ***, *P* < 0.001).

To determine whether EC/11770 has anti-NTM activity *in vivo*, we examined whether this compound was active against M. abscessus in a murine infection model (38). NOD.CB17-*Prkdc^scid^*/NCrCrl (NOD SCID) mice were infected intranasally with M. abscessus subsp. *abscessus* K21. On day 1 postinfection, the lung bacterial burden reached 6.5 × 10^6^ CFU (Fig. 3A). Starting on day 1, 10 mg/kg EC/11770, 250 mg/kg clarithromycin, or drug-free vehicle was administered orally to mice once daily for 10 days. In mice given the drug-free vehicle control, the lung bacterial burden remained unchanged after 10 days (Fig. 3A, day 11). Treatment with EC/11770 at 10 mg/kg achieved a statistically significant 1.5-log reduction in lung CFU, superior though not statistically significantly so to that of clarithromycin at 250 mg/kg (Fig. 3A). We observed a similar pattern of CFU reduction in the spleen (Fig. 3B). Thus, EC/11770 demonstrated efficacy against M. abscessus in a preclinical mouse infection model.

## DISCUSSION

To fast-track *de novo* NTM drug discovery, we sought to test advanced TB active compounds for anti-NTM activity. We therefore tested the benzoxaborole GSK656 and its close analog EC/11770, which both display submicromolar growth inhibitory activity against M. tuberculosis. Interestingly, we found that GSK656 was only active against M. abscessus and inactive against M. avium, whereas EC/11770 showed *in vitro* potency against a collection of M. abscessus and M. avium strains covering a range of subspecies. Critically, EC/11770 demonstrated attractive efficacy in our M. abscessus mouse model, paving the way for this compound to be pursued as a preclinical candidate for NTM. Thus, EC/11770 provides proof-of-principle for *de novo* NTM drug discovery starting from TB actives. With EC/11770’s anti-NTM activity, we also established LeuRS as an NTM drug target. This allows other benzoxaboroles to be considered potential NTM drug candidates, further enabling development of antibiotics for NTM infections.

In contrast to EC/11770’s broad anti-NTM activity, it was somewhat surprising that the structurally similar GSK656 (Fig. 1) was not active against M. avium. The disparity in anti-NTM potency of these two benzoxaboroles could reflect differences in their ability to bind to different mycobacterial LeuRS homologs. Indeed, a recent report that GSK656 is inactive against M. avium suggested that this compound’s activity against M. abscessus may be due to the greater sequence similarity between the LeuRS homologs of M. abscessus and M. tuberculosis (43). Interestingly, the differences between the LeuRS editing domains of M. avium versus those of M. tuberculosis and M. abscessus occur not in the active site but in neighboring amino acid residues that may alter or restrict access to the benzoxaborole-tRNA^Leu^ adduct binding pocket (Fig. S1) (43). The higher potency of GSK656 against M. abscessus compared to that against M. avium was also unusual since dual M. tuberculosis*-*M. abscessus hits are more likely to be M. avium hits than vice versa (18). Our observation that two closely related members of the same drug class can have differential anti-NTM potency shows that an empirical approach is still required to identify a broadly active NTM compound from a drug class with reported TB activity. It also suggests that TB drug development programs should incorporate testing for anti-NTM potency early in the flowchart if the aim is to achieve broad antimycobacterial activity.

EC/11770 was active across the M. abscessus complex, which remains the most difficult NTM infection to treat, with no reliable cure (15). EC/11770 was also potent across the M. avium complex, which is the most commonly isolated NTM lung pathogen (9). Within the M. avium complex, EC/11770 was active against *M. chimaera*, an emerging NTM pathogen associated with cardiac surgery (44). Thus, EC/11770 is well positioned to treat a range of clinically relevant NTM infections. Combined with the fact that EC/11770 was active against M. tuberculosis, this NTM active compound also has the potential to be a pan-antimycobacterial agent.

As observed previously and in this study (40), macrolides used in NTM treatment lose potency against M. abscessus growing as a biofilm *in vitro*. The inability of current NTM drugs to effectively target bacterial biofilms is thought to contribute to poor clinical outcomes in the treatment of NTM infections (42). In this context, EC/11770’s ability to retain its activity against M. abscessus biofilms is significant, and its inclusion in NTM drug regimens could improve efficacy.

As EC/11770 demonstrated *in vivo* efficacy in the M. abscessus mouse infection model, this compound can now be considered for further development as a treatment for NTM infections. Notably, the low (10 mg/kg) dose of EC/11770 was sufficient to achieve a CFU reduction on par with that of a 250 mg/kg dose of clarithromycin, a common macrolide used for NTM infections. Thus, EC/11770 may provide an alternative to macrolide-based NTM treatment regimens, which face both intrinsic and acquired resistance in NTM (11, 45, 46). Rapid emergence of drug resistance has been reported for another benzoxaborole tested as a treatment for urinary tract infections (47). While studies on clinical mycobacterial resistance to benzoxaboroles are lacking, the frequency of resistance for EC/11770 in M. abscessus is 100-fold lower than that observed for other benzoxaboroles in M. tuberculosis (28). Combined with the fact that NTM treatment involves multidrug chemotherapy (8, 9), we expect the risk of developing benzoxaborole resistance in NTM to be greatly reduced.

In conclusion, we screened advanced TB actives for NTM activity and identified EC/11770 as a broad-spectrum anti-NTM compound, introducing a new drug class (benzoxaboroles) and drug target (LeuRS) for NTM drug discovery. Broad microbiological profiling, pharmacokinetic, and efficacy data establish EC/11770 as a promising preclinical candidate for NTM. This study also provides proof of concept that *de novo* NTM drug discovery starting with TB actives is an efficient approach, offering new hope for treating this class of bacterial infections.

## MATERIALS AND METHODS

### Bacterial strains, culture media, and drugs.

M. abscessus Bamboo was isolated from the sputum of a patient with amyotrophic lateral sclerosis and bronchiectasis and was provided by Wei Chang Huang, Taichung Veterans General Hospital, Taichung, Taiwan. M. abscessus Bamboo whole-genome sequencing showed that the strain belongs to M. abscessus subsp. *abscessus* and harbors an inactive clarithromycin-sensitive *erm*(41) C28 sequevar (31, 48). M. avium 11 was isolated from the bone marrow of a patient with AIDS with disseminated infection and was provided by Jung-YienChien and Po-Ren Hsueh, National Taiwan University Hospital, Taipei. Whole-genome sequencing showed that the strain belongs to M. avium subsp. *hominissuis* (32).

Mycobacterium abscessus subsp. *abscessus* strain ATCC 19977, harboring the inducible clarithromycin resistance-conferring *erm*(41) T28 sequevar (49), was purchased from the American Type Culture Collection (ATCC). Mycobacterium abscessus subsp. *bolletii* CCUG 50184T, harboring the inducible clarithromycin resistance-conferring *erm*(41) T28 sequevar (50), and Mycobacterium abscessus subsp. *massiliense* CCUG 48898T, harboring the nonfunctional *erm*(41) deletion sequevar (51), were purchased from the Culture Collection University of Goteborg (CCUG). M. tuberculosis H37Rv ATCC 27294 and M. intracellulare ATCC 13950 were purchased from the ATCC, and *M. chimaera* CCUG 50989T was purchased from the CCUG.

Clinical isolates covering the M. abscessus complex (M9, M199, M337, M404, M422, M232, M506, M111) were provided by Jeanette W. P. Teo (Department of Laboratory Medicine, National University Hospital, Singapore). The subspecies and *erm*(41) sequevars of these isolates were determined previously (37). M. abscessus subsp. *abscessus* K21 was isolated from a patient and provided by Sung Jae Shin (Department of Microbiology, Yonsei University College of Medicine, Seoul, South Korea) and Won-Jung Koh (Division of Pulmonary and Critical Care Medicine, Samsung Medical Center, Seoul, South Korea). This strain harbors the inactive, clarithromycin-sensitive *erm*(41) C28 sequevar as determined previously (38).

For general bacteria culturing and certain MIC experiments, Middlebrook 7H9 broth (BD Difco) was supplemented with 0.5% albumin, 0.2% glucose, 0.085% sodium chloride, 0.0003% catalase, 0.2% glycerol, and 0.05% Tween 80. Unless otherwise stated, solid cultures were grown on Middlebrook 7H10 agar (BD Difco) supplemented with 0.5% albumin, 0.2% glucose, 0.085% sodium chloride, 0.5% glycerol, 0.0003% catalase, and 0.006% oleic acid. Cation-adjusted Mueller-Hinton (CAMH) broth was prepared by first preparing Mueller-Hinton broth (Oxoid CM0405) according to the manufacturer’s instructions and then supplementing aseptically with sterile solutions of CaCl_2_ and MgSO_4_ to achieve CLSI-recommended divalent cation levels (Ca^2+^, 25 mg/liter; Mg^2+^, 12.5 mg/liter).

EC/11770 and GSK656 were provided by GlaxoSmithKline. The synthesis of EC/11770 is described in patent WO 2015021396 (example 10, page 70). Clarithromycin was purchased from Sigma-Aldrich (C9742). All drugs were prepared as 10 mM stocks in 100% dimethyl sulfoxide (DMSO).

### MIC assay in 96-well plate format.

Unless otherwise stated, MIC determination was carried out in 96-well plate format as previously described (18, 37). 96-well plates were initially set up with 100 μl of 7H9 per well. For each compound, a 10-point 2-fold dilution series starting at twice the desired highest concentration was dispensed onto the 96-well plates using a Tecan D300e Digital Dispenser, with the DMSO concentration normalized to 2%. A bacteria culture grown to mid-log-phase (optical density at 600 nm [OD_600_] = 0.4 to 0.6) was diluted to OD_600_ = 0.1 (1 × 10^7^ CFU/ml). 100 μl of the resulting bacteria suspension was dispensed onto the 96-well plates containing compounds to give a final volume of 200 μl per well with an initial OD_600_ = 0.05 (5 × 10^6^ CFU/ml) and final DMSO concentration of 1%. Final compound concentration ranges were typically 50 to 0.098 μM or 6.25 to 0.012 μM but were adjusted to 100 to 0.195 μM for testing of EC/11770-resistant mutant strains. Untreated control wells were included on each plate that contained bacteria suspension and 1% DMSO. Plates were sealed with parafilm, stored in boxes with wet paper towels, and incubated at 37°C with shaking (110 rpm). Plates were incubated for 3 days (M. abscessus complex), 4 days (M. avium complex), or 7 days (M. tuberculosis). To determine growth, OD_600_ was measured using a Tecan Infinite M200 plate reader on day 0 and day 3, 4, or 7. Two biological replicates were performed. Clarithromycin (M. abscessus and M. avium complexes) or Rifampin (M. tuberculosis) were included in each experiment as a positive control.

For each well on the 96-well plate, bacterial growth was calculated by subtracting the day 0 OD_600_ value from the endpoint (day 3, 4, or 7) OD_600_ value. For each compound series, the bacterial growth values for the untreated control wells were averaged to give the average drug-free bacterial growth. For compound-containing wells, percentage growth was calculated by dividing their growth values by the average drug-free bacterial growth for the compound series and multiplying by 100. For each compound series, we plotted percentage growth versus compound concentration. By visual inspection of the dose-response curve, we determined the MIC of a compound as the compound concentrations that would result in 90% growth inhibition.

For MIC determination in CAMH broth, experiments were set up as described above with the following changes. Compounds were dispensed onto 96-well plates with 100 μl of CAMH broth per well. A mid-log-phase bacteria culture (initially in 7H9) was washed once and resuspended with CAMH broth. The culture was then diluted to OD_600_ = 0.1 (1 × 10^7^ CFU/ml) using CAMH broth before dispensing to the 96-well plates.

### MIC and MBC determination in culture tubes.

M. abscessus Bamboo culture was grown to mid-log-phase (OD_600_ = 0.4 to 0.6) and diluted to OD_600_ = 0.1 (1 × 10^7^ CFU/ml). Aliquots of 1.2 ml of the resulting bacteria suspension were transferred into 14 ml vented, round-bottom tubes (Thermo Fisher 150268, Rochester, NY, United States). A 10-point 2-fold dilution series of the compound was prepared, starting at 100 times the desired highest concentration. The compound concentration range tested was 100 to 0.195 μM. For each drug concentration tested, 12 μl of drug stock was added to two tubes and vortexed. Aliquots of 12 μl of DMSO were added to two tubes as the untreated controls (1% final DMSO concentration). From each tube, 200 μl was transferred to wells on a 96-well plate and the OD_600_ was measured using a Tecan Infinite M200 plate reader (day 0 reading). The tubes (1 ml final volume) were incubated on a tilted rack at 37°C on an orbital shaker at 220 rpm. On day 2, tubes were vortexed before transferring 200 μl onto a 96-well plate to measure the OD_600_ again (day 2 reading). To determine the MIC, day 0 and day 2 OD_600_ values were analyzed as previously described for MIC determination in 96-well plate format. To determine the MBC, CFU measurement was done for the OD_600_ = 0.1 bacteria suspension on day 0 and for each tube on day 2. Specifically, serial 10-fold dilutions were prepared in phosphate-buffered saline (Thermo Fisher 10010023) containing 0.025% Tween 80 (PBS/Tween 80) and plated on 7H10 agar. The MBC was defined as the lowest concentration of drug that reduced the CFU/ml value by 10-fold relative to the day 0 CFU/ml value.

### Biofilm growth inhibition assay.

The biofilm growth inhibition assay was performed as previously described (40). Innovotech MBEC 96-well biofilm assay plates (Innovotech 19111, Edmonton, AB, Canada) were used, and the supplier’s manual was followed with minor modifications. Mid-log-phase M. abscessus Bamboo precultures (OD_600_ = 0.4 to 0.6) were spun down at 3200 × *g* for 10 min at 25°C and washed with 7H9 medium without Tween 80 (7H9nt). Bacteria were resuspended into 25 ml 7H9nt to an OD_600_ of 0.0125 (1.0 × 10^6^ CFU/ml). A total of 150 μl of bacteria suspension was dispensed into each well of MBEC multititer plates, and the polystyrene protrusions (pegs) of the MBEC lid were inserted into the culture-containing wells for 24 h at 37°C on an orbital shaker at 110 rpm to allow attachment of the bacteria to the pegs and initiation of biofilm growth. The lids with the pegs were transferred to a new MBEC multititer plate containing 150 μl of fresh 7H9nt medium per well without bacteria (0 h time point). After that, the pegs with growing biofilm were transferred once a day to a new multititer plate containing fresh 7H9nt medium. To measure growth of the biofilm formed on the peg, the pegs were washed in 200 μl of 7H9nt medium before they were aseptically removed and placed in 1.7 ml microcentrifuge tubes (VWR 87003-294, Rador, PA, United States) containing 500 μl PBS/Tween 80. The microcentrifuge tubes were vigorously vortexed at 2,000 rpm for 90 s at 25°C to detach the bacteria from the pegs before samples were serially diluted and plated for the determination of CFU/peg. To determine the biofilm MIC of antibiotics, appropriate drug concentrations or DMSO (untreated control, 1% final concentration) were added at the 24 h time point, and CFU were determined after 48 h of incubation with antibiotic (72 h time point). The average drug-free biofilm growth was calculated by subtracting the average 24 h CFU/peg value from the average 72 h CFU/peg value for the untreated control pegs. The biofilm MIC was defined as the lowest drug concentration that reduced the CFU/peg by 90% relative to that of the average drug-free biofilm growth.

The biofilm MBC was defined as the concentration of drug that reduced the CFU/peg by 10-fold relative to the CFU/peg at 24 h.

### Selection of spontaneous resistant mutants.

Spontaneous resistant mutants were selected as described previously (52). Exponentially growing M. abscessus Bamboo culture (10^7^ to 10^9^ CFU) was plated on 7H10 agar containing 100 μM EC/11770. The plates were incubated for 7 days at 37°C. Apparent resistant colonies were purified and confirmed by restreaking on agar containing the same concentration of drug. Two independent batches of resistant mutants were generated in this manner. Genomic DNA was extracted as described previously using the phenol-chloroform method (53). Sanger sequencing of the *leuS* (*MAB_4923*) genomic region was performed by Genewiz (Genewiz, Inc., South Plainfield, NJ, USA) using four primers (leuS-UpStrm-Fwd, 5′-GTCCCGAAGTTAATAACCGC-3′; leuS-Int-Fwd, 5′-GACGCAGTGGATTTTCCTAC-3′; leuS-Int-Rev, 5′-AGGCTCTTTCCGATCTTCCC-3′; leuS-DwnStrm-Rev, 5′-AGAACTCACCGAACATGAAG-3′). Based on the domain map of LeuRS in M. tuberculosis (28), we identified the editing domain of M. abscessus LeuRS as amino acid residues V292 to K502 (nucleotides 874 to 1506) and determined that all spontaneous resistant mutants possessed a missense mutation in this region.

### Kinetic aqueous solubility assay.

The aqueous solubility of test compounds was measured using an in-house method utilizing quantification via chemiluminescent nitrogen detection (CLND): 5 μl of 10 mM DMSO stock solution was diluted to 100 μl with pH 7.4 phosphate-buffered saline, equilibrated for 1 h at room temperature, and filtered through Millipore Multiscreen HTS-PCF filter plates (MSSL BPC). The eluent is quantified by a suitably calibrated flow injection chemiluminescent nitrogen detection (CLND or CAD). This assay has a dynamic range between the lower detection limit of 1 and 500 μM, governed by the protocol’s 1:20 dilution into pH 7.4 phosphate buffer solution from nominal 10 mM DMSO stock.

### ChromlogD assay.

The Chromatographic Hydrophobicity Index (CHI) values are measured using reversed phase high-performance liquid chromatography (HPLC) column (50 by 2 mm 3 μM Gemini NX C18, Phenomenex, UK) with fast acetonitrile gradient at starting mobile phase of pHs 2, 7.4, and 10.5. CHI values are derived directly from the gradient retention times by using a calibration line obtained for standard compounds. The CHI value approximates to the volume percent organic concentration when the compound elutes. CHI is linearly transformed into ChromlogD by least-square fitting of experimental CHI values to calculated ClogP values for over 20,000 research compounds using the following formula: ChromlogD = (0.0857 × CHI) − 2.00. The average error of the assay is ±3 CHI unit or ±0.25 ChromlogD.

### AMP (artificial membrane permeability) assay.

An 8% l-α-phosphatidylcholine (EPC) in 1% cholesterol decane solution and a 1.8% EPC in cholesterol decane solution were prepared. The lipid solution was then aliquoted into 4 ml capped vials, sealed with parafilm, and stored in a −20°C freezer. The lipid solution was then transferred from 4 ml vial into a 96-well half area plate (130 μl/well) for daily assay usage. An additional 50 mM phosphate buffer with 0.5% encapsin (pH 7.4) was prepared. The assay was run by the Biomek FX and Biomek software. The assay procedure is written under the Biomek software. For one batch assay, it can test two 96-well sample plates with at least one standard on each sample plate. The total assay time was about 4 h. A total of 3.5 μl of lipid solution was added to the filler plate and shaken for 12 s, 250 μl of buffer was added to the donor side, and 100 μl of buffer was added to the receiver side. The assay plate was shaken for 45 min before adding the compounds. The test compounds (2.5 μl) were added to the donor side. The assay was run as replicates: assay plates 1 and 2 tested sample plate 1, and assay plates 3 and 4 tested sample plate 2. The assay plates were then incubated and shaken for 3 h at room temperature. The assay samples were transferred to the HPLC analysis plates, and 100 μl of receiver solution was aspirated and transferred to the receiver for analysis. Similarly, another 100 μl from the donor solution was transferred to the donor analysis plate. Compound concentration was measured by HPLC at different time points.

### Stability in microsomes.

Intrinsic clearance (CLi) values were determined in mouse and human liver microsomes. Test compounds (final concentration 0.5 μM) were incubated at 37°C for 30 min in 50 mM potassium phosphate buffer (pH 7.4) containing 0.5 mg microsomal protein/ml. The reaction was started by addition of cofactor NADPH. At 0, 5, and 20 min, an aliquot (90 μl) was taken, quenched with acetonitrile-methanol containing an appropriate internal standard, centrifuged, and analyzed by liquid chromatography-tandem mass spectrometry (LC-MS/MS). The intrinsic clearance (CLi) was determined using the following equation: CLi = *k*(ml of incubation/mg microsomal protein) × (mg microsomal protein/g liver)where *k* is the turnover rate constant of the ln(% remaining compound) versus time regression and mg microsomal protein/g liver is 52.5 for both mouse and human.

### Pharmacokinetics studies.

For pharmacokinetic studies, CD-1 male mice (22 to 25 g) were used for intravenous route and C57BL/6 female mice (18 to 20 g) were used for oral route. All animal studies were ethically reviewed and carried out in accordance with European Directive 2010/63/EU and the GSK policy on the care, welfare, and treatment of animals.

EC/11770 was administered by intravenous route at 1 mg/kg single dose in saline and by oral gavage at 1 mg/kg and 10 mg/kg single doses in 1% methyl cellulose (1% MC). Aliquots of 20 μl of blood were taken from the lateral tail vein by puncture from each mouse (*n* = 3 per route and dose) at 5, 15, and 30 min 1, 2, 4, 6, 8, and 24 h postdose for intravenous route and at 15, 30, and 45 min 1, 2, 4, 6, 8, and 24 h postdose for oral route. The blood aliquots were mixed with 40 μl of water and stored at −80°C prior to analysis. The samples were processed by protein precipitation: aliquots of 20 μl were mixed with acetonitrile/methanol (80:20 vol/vol) and then filtered in 0.45 μm well-plates (hydrophobic polytetrafluorethylene [PTFE], Millipore). The filtrates were analyzed by UPLC-MS/MS for the establishment of compound concentration. The ultraperformance liquid chromatography (UPLC)-MS/MS system included a UPLC Acquity (Waters) and an API4000 mass spec. (AB Sciex). A volume of 7.5 μl of sample was injected into the system, with an UPLC solvent gradient of 2 min and with a positive multiple reaction monitoring (MRM) mass detection mode.

Pharmacokinetic analysis was performed by noncompartmental data analysis (NCA) with Phoenix WinNonlin 6.3 (Pharsight, Certara L.P), and supplementary analysis was performed with GraphPad Prism 6 (GraphPad Software, Inc.).

### M. abscessus mouse infection model.

*In vivo* efficacy determinations were carried out as described previously, using 8-week-old female NOD.CB17-*Prkdc^scid^*/NCrCrl (NOD SCID) mice (Charles River Laboratories) and the M. abscessus subsp. *abscessus* K21 strain (38). Briefly, anesthetized animals were infected by intranasal delivery of ∼10^6^ CFU of M. abscessus subsp. *abscessus* K21. Acute infection was achieved within 1 day. Drugs or the vehicle control were administered to NOD SCID mice once daily for 10 consecutive days by oral gavage, starting from 1 day postinfection. Clarithromycin was formulated in 0.4% methyl cellulose-sterile water and administered at a dose of 250 mg/kg. EC/11770 was formulated in 0.4% methyl cellulose-sterile water and administered at 10 mg/kg. All mice were euthanized 24 h after the last dose (11 days postinfection), and their lungs and spleen were aseptically removed prior to homogenization. The bacterial load in these organs was determined by plating serial dilutions of the organ homogenates onto Middlebrook 7H11 agar (BD Difco) supplemented with 0.2% (vol/vol) glycerol and 10% (vol/vol) oleic acid-albumin-dextrose-catalase (OADC). The agar plates were incubated for 5 days at 37°C prior to counting of colonies. All studies were conducted in accordance with the GSK policy on the care, welfare, and treatment of laboratory animals and were reviewed by the Institutional Animal Care and Use Committee either at GSK or by the ethical review process at the institution where the work was performed. All experiments involving live animals were approved by the Institutional Animal Care and Use Committee of the Center for Discovery and Innovation, Hackensack Meridian Health.

### Sequence analysis of mycobacterial LeuRS editing domains.

Amino acid sequences of LeuRS from M. tuberculosis H37Rv ATCC 27294, M. abscessus Bamboo, and M. avium 11 were aligned using Clustal Omega (https://www.ebi.ac.uk/Tools/msa/clustalo). The alignment was formatted using ESPript 3.0 (http://espript.ibcp.fr/) (54).

## Supplementary Material

Supplemental file 1
