# Supplementary material for: A Leucyl-tRNA Synthetase Inhibitor with Broad-Spectrum Antimycobacterial Activity
Source: Antimicrob Agents Chemother. 2021 Apr 19;65(5):e02420-20. doi: 10.1128/AAC.02420-20 (PMC8092876; doi:10.1128/AAC.02420-20)
Supplement: Supplemental file 1 [file AAC.02420-20-s0001.pdf]

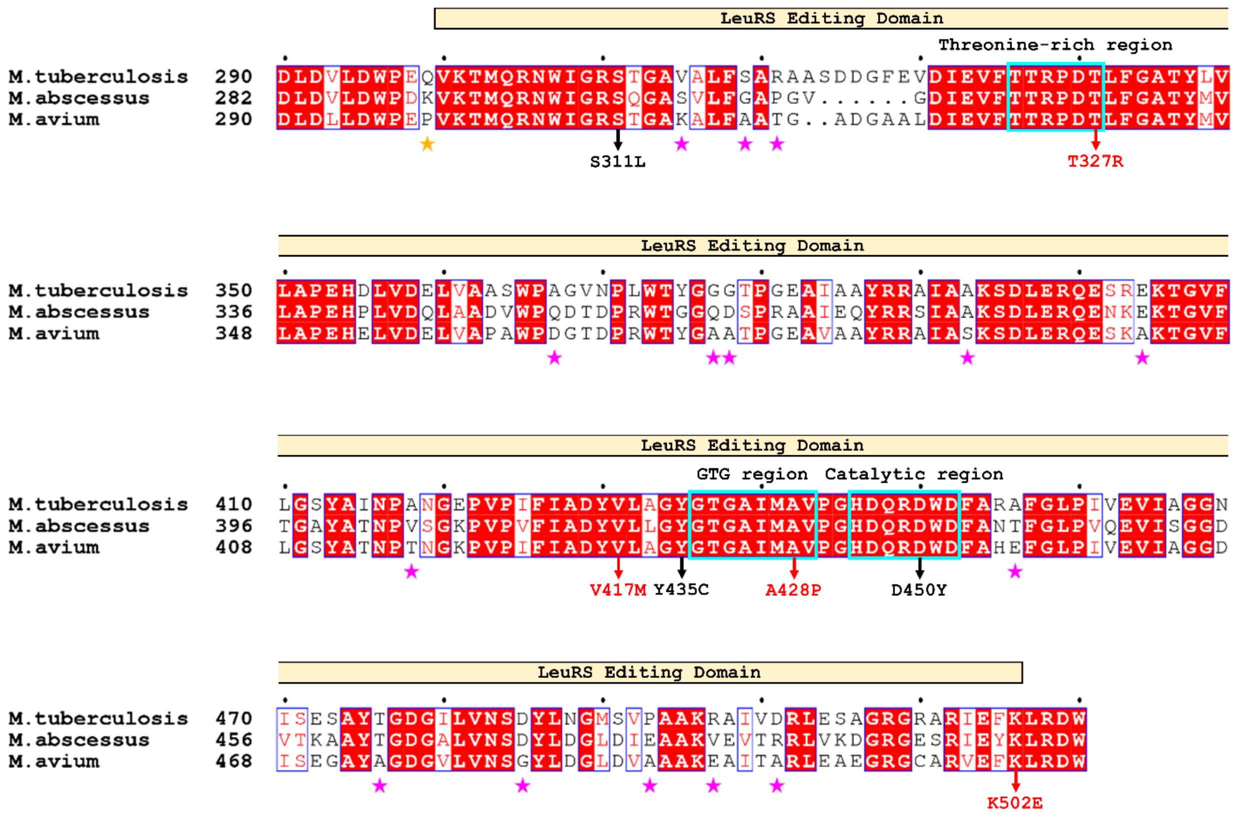

Figure S1. Sequence alignment of mycobacterial LeuRS editing domains. The amino acid sequences of LeuRS from *M. tuberculosis* H37Rv, *M. abscessus* Bamboo and *M. avium* 11 were aligned using Clustal Omega. A portion of the alignment encompassing the LeuRS editing domain (*M. tuberculosis* LeuRS residues 300-516) is displayed. The threonine-rich region, GTG region and catalytic region (cyan boxes) are key components of the LeuRS editing active site (1, 2) and are conserved across all three species. Fifteen residue differences (purple stars) that distinguish the *M. avium* 11 LeuRS editing domain from those of *M. tuberculosis* H37Rv and *M. abscessus* Bamboo were identified. Three of these residue differences (*M. tuberculosis* LeuRS T476, P493 and R497) are positioned near the entrance of the binding pocket for the benzoxaborole-tRNA^{Leu} adduct (3). An additional residue difference that occurs outside of the LeuRS editing domain (*M. tuberculosis* LeuRS Q299, orange star) is close to the tRNA^{Leu}-LeuRS interface (3). Mutations identified in benzoxaborole-resistant mutants from *M. tuberculosis* H37Rv (black arrows)(4) and *M. abscessus* Bamboo (red arrows, Table 4) are also indicated.

REFERENCES

1. Lincecum TL, Jr., Tukalo M, Yaremchuk A, Mursinna RS, Williams AM, Sproat BS, Van Den Eynde W, Link A, Van Calenbergh S, Grotli M, Martinis SA, Cusack S. 2003. Structural and mechanistic basis of pre- and posttransfer editing by leucyl-tRNA synthetase. Mol Cell 11:951-63.
2. Pang YL, Martinis SA. 2009. A paradigm shift for the amino acid editing mechanism of human cytoplasmic leucyl-tRNA synthetase. Biochemistry 48:8958-64.
3. Dong W, Li S, Wen S, Jing W, Shi J, Ma Y, Huo F, Gao F, Pang Y, Lu J. 2020. In Vitro Susceptibility Testing of GSK656 against Mycobacterium Species. Antimicrob Agents Chemother 64:e01577-19.
4. Palencia A, Li X, Bu W, Choi W, Ding CZ, Easom EE, Feng L, Hernandez V, Houston P, Liu L, Meewan M, Mohan M, Rock FL, Sexton H, Zhang S, Zhou Y, Wan B, Wang Y, Franzblau SG, Woolhiser L, Gruppo V, Lenaerts AJ, O'Malley T, Parish T, Cooper CB, Waters MG, Ma Z, Ioerger TR, Sacchettini JC, Rullas J, Angulo-Barturen I, Perez-Herran E, Mendoza A, Barros D, Cusack S, Plattner JJ, Alley MR. 2016. Discovery of Novel Oral Protein Synthesis Inhibitors of Mycobacterium tuberculosis That Target Leucyl-tRNA Synthetase. Antimicrob Agents Chemother 60:6271-80.
